# The risk-risk trade-offs: Understanding factors that influence women’s decision to use substances to boost breast milk supply

**DOI:** 10.1371/journal.pone.0249599

**Published:** 2021-05-03

**Authors:** Gabriella Zizzo, Lisa H. Amir, Vivienne Moore, Luke E. Grzeskowiak, Alice R. Rumbold

**Affiliations:** 1 Faculty of Health and Medical Sciences, The University of Adelaide, Adelaide, South Australia, Australia; 2 Judith Lumley Centre, La Trobe University | Royal Women’s Hospital, Parkville, Victoria, Australia; 3 Faculty of Health and Medical Sciences, The University of Adelaide, South Australia | Women and Kids, South Australian Health and Medical Research Institute, South Australia, Australia; 4 Women and Kids, South Australian Health and Medical Research Institute, South Australia, Australia; University of North Carolina at Greensboro, UNITED STATES

## Abstract

Galactagogues are foods, herbs or medications thought to support or increase breast milk supply. The use of galactagogues during lactation is becoming increasingly common despite limited evidence regarding effectiveness and safety, and no definitive recommendations for use in clinical practice. The aim of this study is to explore factors influencing women’s decisions to use galactagogues during lactation. Twenty-two semi-structured interviews were conducted in October and November 2019 (over the telephone or in person) with participants located in most Australian states and territories, including metro and regional areas. Interviews were audio-recorded, transcribed verbatim and thematically analysed using NVivo. Analysis revealed that following a concern about breast milk supply, the decision to use galactagogues was influenced by three core and interrelated domains: access to and quality of breastfeeding support, maternal agency and determination to provide breast milk. Women revealed many problematic experiences with health care professionals that left them feeling dismissed and confused due to provision of inconsistent and insufficient information that was sometimes at odds with their desire to provide breast milk. In this instance, some women turned to galactagogues to regain agency. A range of broader dimensions influencing decision-making also emerged. These were separated into categories that emphasise distinctions relating to breast milk supply, which included: maternal emotional wellbeing, social norms and pressures, concerns about infant development, maternal physical health and lactation history, as well as those relating specifically to galactagogue use, including: desire for a guaranteed/urgent response, risk-risk trade-off, acceptance and trust, and accessibility and cost. In understanding the complexity of decision-making concerning these substances, we identify opportunities to improve breastfeeding counselling and support. We recommend that support be individually tailored to manage conflicting information, adopt communication styles that encourage trust and processes that enable shared decision-making to enhance or restore maternal agency. There is also considerable need to address evidence gaps regarding the effectiveness and safety of commonly used galactagogues, so that women can be appropriately counselled about potential benefits and harms.

## Background

Supporting breastfeeding is a public health priority, based on the well-established benefits of breastfeeding for maternal and child health [1,2]. Although there are very high rates of initiation of breastfeeding worldwide (>80% of mothers in most countries), rates of breastfeeding decline markedly in the first few months after birth [2], most notably in high-income countries. This occurs for a multitude of reasons, reflecting breastfeeding difficulties and insufficient access to lactation support and postnatal care, as well as broader family and social support, access to paid maternity leave and social and cultural norms about infant feeding [3]. For some women, ceasing breastfeeding earlier than initially desired leaves them feeling stigmatised [4] and can be a source of shame, grief, guilt, distress and anxiety [5,6], contributing to a reduced sense of agency to achieve breastfeeding goals and poor psychological wellbeing [7,8]

One of the most common reasons cited for early cessation of breastfeeding is perceived low or insufficient milk supply [9–12]. This should be managed initially by the provision of breastfeeding counselling and support [13], however, when concerns persist, substances known as galactagogues are often recommended [13,14]. Galactagogues are foods, herbal supplements or medications believed to assist initiation, maintenance, or augmentation of breast milk supply [15]. For centuries, women have used herbal or food preparations such as fenugreek, fennel, oats and brewer’s yeast and other local botanicals, in this way [16–18].

Recently, there has been an increase in the direct marketing of herbal galactagogues and food and beverage products such as ‘lactation cookies’ and breastfeeding teas/tisanes [19]. In addition, a growing number of pharmacological agents to support lactation have been evaluated in clinical trials [14]. Of these, the medication domperidone is the most widely studied, and has been shown to result in modest increases in breast milk volume among mothers who have given birth prematurely–a group at high risk of lactation difficulties [20]. However, the generalisability of these findings to all women who give birth is unclear and there are ongoing concerns about rare but serious adverse effects associated domperidone, including cardiac arrhythmias [21]. Such concerns have led to regulatory warnings against its use during lactation by the Food and Drug Administration in the United States [15,22].

Evidence about the safety and efficacy of herbal and food products marketed as galactagogues is inconsistent, with both positive and negative impacts on milk supply reported for some products (e.g. fenugreek), as well possible maternal and infant side effects [15,16]. As a result of inconclusive evidence, peak professional societies such as the Academy of Breastfeeding Medicine, do not recommend use of any specific galactagogues during lactation [15,22].

Despite limited evidence to support use, current research indicates that the number of women using galactagogue is increasing [23–26]. In high- and middle-income countries, prescribed pharmacological galactagogues are now a common treatment for insufficient milk supply [19,27–29]. In a recent study of over 200,000 mothers in British Columbia, Canada, 1 in 5 were prescribed domperidone in the first six months after birth, with reported use doubling between 2002 and 2011 [29]. While information about utilisation patterns of other galactagogues is limited, a recent US study found that approximately 50% of breastfeeding women reported using a herbal preparation postpartum to increase milk supply [19].

Within academic research, there has been little prior exploration of the reasons why women are increasingly seeking out or being prescribed substances to increase breast milk supply. The limited available research has focussed on herbal galactagogues only [24–26] or has been undertaken in regions where access to pharmaceutical galactagogues is restricted [19]. Nevertheless, the findings indicate that when taking a herbal product, some women report feelings of increased confidence and reassurance in their ability to breastfeed [25,26]. This suggests that there may be important social and emotional aspects surrounding galactagogue use that are not well understood. Further, there is some evidence that women perceive herbal galactagogues to be ‘safer’ than pharmacological agents, and in the absence of clear guidelines for use, appear to be relying on the internet and social media for anecdotal reports about efficacy [19,25,26,30,31].

To date, no studies have examined decision-making about the full range of galactagogue treatment options available. In addition, there has been limited attention paid to the broader factors that may influence decisions about galactagogue use, including cost and accessibility, the level of informal and formal support women engage with, the increased marketing of products (particularly via social media), the degree of social expectations for mothers to provide breast milk [30–32]. The latter is important as some women who stop breastfeeding express feelings of ‘failure’, illustrating how breastfeeding is intrinsically linked to ideals about ‘good mothering’ and is often framed as a moral ‘choice’ and responsibility [33,34]. Thus, pressure to breastfeed and the resulting influence on psychological wellbeing and diminishing of maternal agency may well be contributing to use of galactagogues. The aim of this paper was to undertake an in-depth exploration of the key factors influencing decision-making around the use of a broad range of galactagogues.

## Methods

Participants in this study were recruited from a large, national online survey of Australian women’s awareness and use of galactagogues to boost breast milk supply. The survey was promoted through various social media platforms including member pages for the Australian Breastfeeding Association (the leading, national breastfeeding advocacy and mother-to-mother support organisation in Australia) and the Miracle Babies Foundation (an Australian organisation supporting premature and sick newborns, their families and the hospitals that care for them). After anonymously completing the original survey online, women were invited to express their interest in participating in an interview and provide their contact details. The inclusion criteria were intentionally broad, including women who had challenges breastfeeding and/or with supply and women who did not. Participants were recruited from all states and territories however those living outside Australia were excluded. Participants were given the option of participating in person, over the telephone or via video conferencing software. Ethical approval for the project was granted by University of Adelaide Human Research Ethics Committee (Approval number H-2019-167).

Semi-structured interviews were conducted during October and November of 2019 by an interviewer located in Adelaide, South Australia. To align with ethical protocols, prior to the interviews Information Sheets and Consent Forms were distributed providing details including information about audio recording, transcription, data storage and de-identification processes. Signed consent forms were collected at the time of the interview, Author 1 (who conducted all the interviews) confirmed consent and asked permission to begin the audio recording. Following the interview, participants were offered an AUD$30 gift card in acknowledgement of their time.

To encourage participants to discuss the topics or events they considered important to their decision-making about galactagogues interviews followed a semi-structured interview guide (attached). Author 1 conducted all of the interviews. She has a PhD and experience conducting interviews with mothers around breastfeeding. At the time of the project, Author 1 was employed as a Research Fellow. In order to situate the research and the researcher, the interviews began with a preamble and a brief introduction to Author 1 including relevant personal details (family background, age, maternal status). The participants were also told about the researcher’s background in this area of inquiry, which was intended to inform the participants of the researcher’s subject position.

The survey was undertaken between September to December 2019. Over 2,000 women participated and of these, 486 women indicated they may be interested in participating in an interview. We approached women in the order that they completed the survey, with the goal of interviewing approximately 20 women. This entailed contacting the first 46 women who expressed interest, and of these, 22 women replied to confirm their interest and scheduled an interview. The number of interviews included two pilot interviews to test and modify the interview guide. Sixteen interviews were conducted over the telephone, four face-to-face in the local public maternity hospital and two via video conference software. Twenty of the interviews were transcribed by a professional transcription service (pilot interviews were not transcribed), de-identified and assigned pseudonyms.

Interviews were analysed thematically informed by the approach to qualitative data analysis in health-related fields endorsed by Braun and Clarke [35]. Following each interview, the researcher recorded brief memos to document key points including findings that were consistent or contradictory. After the interviews were complete, initial fieldnotes and the semi-structured interview guide were used to generate the early coding framework. A first reading of the interview transcripts (familiarisation stage), was used to refine this coding framework further, which was then checked by another member of the project team (senior author). Using NVivo (version 12) software, the data were coded and classified into core themes and subthemes which were revised further in consultation with the wider project team at two different time periods.

## Findings

The average age of the participants was 34 years (range 29–50), giving birth between 2009 and 2019. Twelve participants reported having a degree level education, eight a higher degree and around a third of participants were employed in a health profession or research role. Ten of the participants were first time mothers, four reported giving birth prematurely and nine reported having a caesarean section. One participant identified as being born outside of Australia (New Zealand), one identified as Aboriginal and Torres Strait Islander (First Nation), and all spoke English as the primary language in the home.

All twenty-two participants initiated breastfeeding, and at the time of the interviews, nine were still breastfeeding or supplying expressed breast milk. Domperidone was the most commonly used pharmaceutical galactagogue (n = 15, 68%), but in most cases was used in combination with a non-pharmaceutical galactagogue. Twenty participants (91%) reported taking at least one non-pharmaceutical substance, including fenugreek, lactation cookies, brewer’s yeast, and various combinations of products.

**Fig 1:**
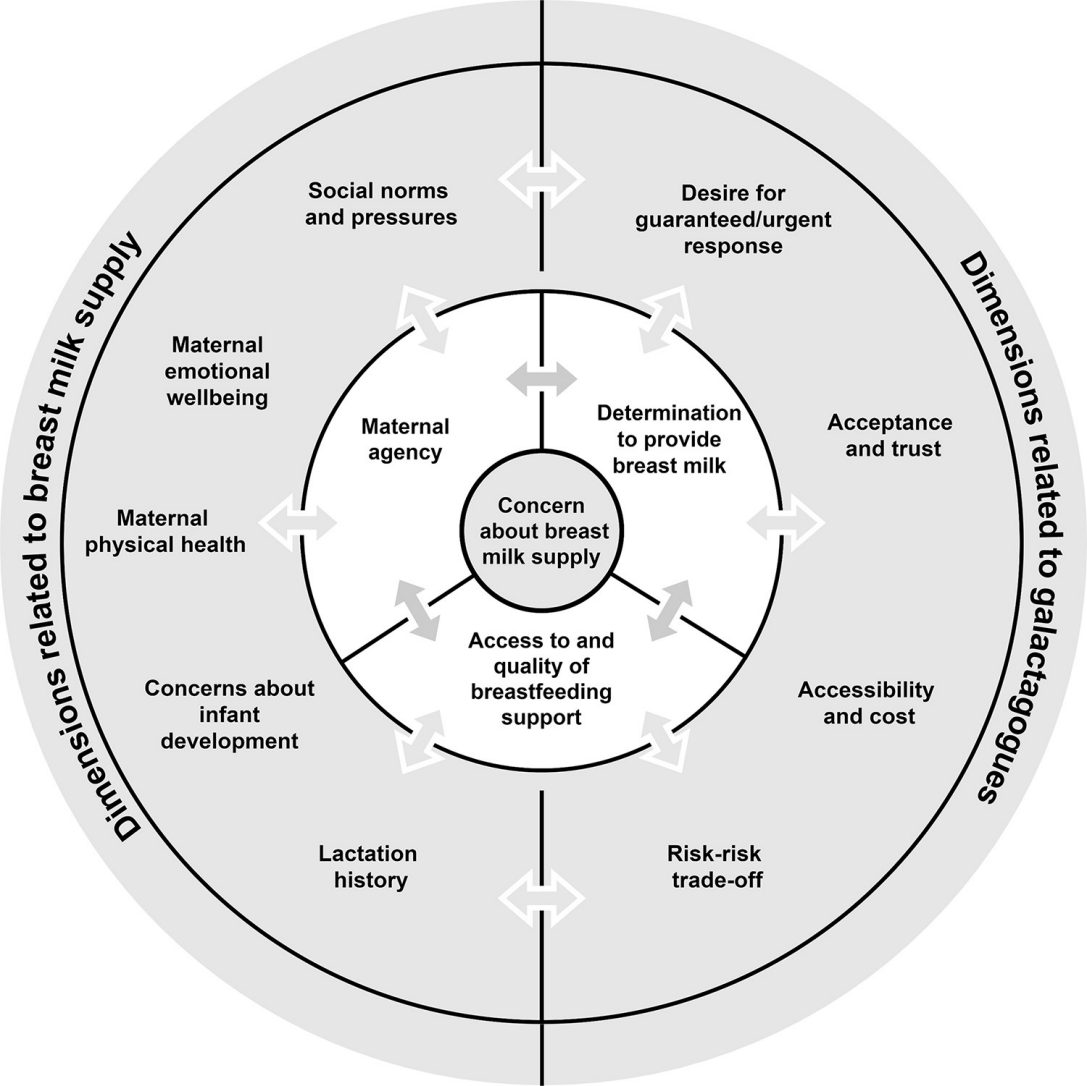
Domains and dimensions influencing women’s decision-making regarding the use of galactagogues.

Fig 1 provides a summary of the core themes that emerged during analysis regarding the factors that influenced women’s decisions to use galactagogues. As depicted, following a trigger–a real or perceived concern about breast milk supply–their decisions to use a galactagogue/s were underpinned by the core domains of: access to and quality of breastfeeding support available, maternal agency, and determination to provide breast milk. Outside of the core domains, important broader dimensions of decision-making were revealed, indicative of the complexity of women’s deliberations concerning galactagogues. These dimensions reflected a range of maternal experiences, external factors and processes that had an overarching influence on decision-making. The dimensions were separated into categories that emphasise distinctions relating to breast milk supply (left of outer ring) which included maternal emotional wellbeing, social norms and pressures, concerns about infant development, maternal physical health and lactation history, as well as distinctions relating to galactagogue use (right of outer ring) which included desire for a guaranteed/urgent response, risk-risk trade-off, acceptance and trust, and accessibility and cost. The bi-directional arrows in Fig 1 are indicative of the dynamic nature of these influences throughout the decision-making process with many factors interrelated. The dimensions were not equal in influence, as the value attributed to each was highly individualised and situational, and many women were drawing on more than one dimension to make their decisions about what to use to boost breast milk supply. These decisions were not final with many recycling through the process of seeking more advice, support or information if the expected outcome did not occur.

### Concern about breast milk supply

Central to the use of galactagogues is the initial trigger that signalled concerns regarding breast milk supply (depicted in Fig 1 as the inner-most ring). Concerns about supply may be real or perceived, could be raised by the individual or by external sources, and may involve a specific diagnosis of lactation insufficiency (however a number of participants discussed ‘self-diagnosis’).

Concerns about supply were often described as arising out of worries about the baby’s weight gain, contents of nappies (diapers), hydration, temperature and baby’s unsettledness. Also discussed were issues around delayed milk production, ‘no letdown’ or ‘not feeling full’ which many women interpreted as impacting their milk supply. Other concerns around breastfeeding and supply included perceptions that the baby was not interested in or refusing the breast, positioning, difficulty with latching or sucking or the appearance of their breast milk. For example, Anita described her breast milk as thin and watery, prompting her to problematise her supply:

I had the thinnest little–and I’m like, I don’t understand why. My baby needs the extra fat and calories and I’ve got the stupidest, like liquidy [sic] milk that you could possibly have. Like, mine’s skim milk.

(Anita, South Australia)

Some women, who were feeding their subsequent babies, expressed concerns based on previous issues with supply, and addressed these concerns with the prophylactic use of galactagogues.

### Access to and quality of breastfeeding support

When concerns regarding breast milk supply arose women actively sought out breastfeeding support, as well as information and advice on how to manage their supply. For many this is when the possibility of using a galactagogue was first introduced.

Based on the information and support received, some women directed their attention to production-increasing techniques. Participants accessed information about various methods to boost supply (including galactagogues) from a variety of sources including breastfeeding clinics, doctors (including general practitioners (GPs), paediatricians, obstetricians), lactation consultants (some but not all identified as International Board-Certified Lactation Consultants [IBCLC], home visiting and in public hospitals), Australian Breastfeeding Association hotline and website, midwives and maternal and child health nurses (home visiting, community, hospital), targeted marketing based on browsing history, online (blogs, Facebook groups, Instagram posts, forums), peers (family, mothers groups) and pharmacists.

Although all participants engaged in some form of breastfeeding support, many discussed the effectiveness and quality of the support and various barriers to accessing appropriate support. For example, the breastfeeding support provided in hospitals was in high demand and at times described as hurried and time restricted. Similarly, due to a high demand on breastfeeding clinics many missed out on responsive support and were left to manage their concerns on their own. As Maria indicated, the breastfeeding clinic she used referred her to an in-patient breastfeeding support service with a long waiting list;

So, I went to an appointment there, and I was referred for a residential stay, which ultimately didn’t happen for about five months or something […] There’s huge waiting lists. So, the nurse who I had the appointment with raised, yeah, Motilium [domperidone] and various other–are they called galactagogues? Yep, so that’s when it was raised to me […] I then went to my GP and I got a script.

(Maria, New South Wales)

Maria, and others like her felt the situation was too urgent to wait months for support so the medication was sought out to fill gaps in access to support. Others filled these gaps by relying on targeted marketing and promotional information linked to commercial products (foods/herbs), viewed as creditable and trusted sources of information about what practices to implement to boost their breast milk.

I did some reading on how to actually get there and then came across [brand of commercial lactation cookies] from there and then [the website] actually explained why each ingredient is in there and how it helps

(Fiona, New South Wales)

### Maternal agency

Women’s sense of maternal agency underpinned their decision-making about using galactagogues and was strongly influenced by the information and support they received from health care professionals and their peers. A variety of accounts were described suggesting that galactagogue use occurs both in the context of loss of agency, as well as to actively maintain agency, enabling women to make decisions to facilitate their breastfeeding goals. For example, some women turned to galactagogues following interactions with health care professionals where their agency was diminished, which they described as feeling dismissed, that their breastfeeding challenges were not believed, their values and goals ignored, and involved the delivery of information they considered unhelpful and delivered judgementally. As Mandy explained, despite her clearly communicated commitment to breastfeed following the birth of her second child; “*one [healthcare professional in the maternity ward] came in to say ‘why are you trying to be a hero*? *It’s not the end of the world*, *just give the baby some formula’”*.

In addition to not being supported to achieve her breast feeding goals, Mandy’s experience exemplified how some women found it difficult to manage the discordant messaging from breastfeeding promotion information that ‘breast is best’ against the attitudes of some health professionals and family/friends that infant formula was just as good as breast milk.

While some women reported lacking confidence to speak with authority concerning their breastfeeding support needs, this was not a universal experience. However, even when women did express confidence in advocating for themselves, some reported feeling undermined. As first-time mother Maria explained, she was confident in her abilities to interact with medical professionals whom she found to be dismissive and patronising:

…people are, I think, sort of quite ready to assume that a first-time mum would be a bit hysterical, and sort of unable to cope, but the crying was just–I just knew. Like, I had lots of experience with babies, and I was very capable of settling her, and you just know there’s a difference with the crying. Like, regular sort of eight, nine, ten-week-old babies don’t cry inconsolably, like scream for hours, you know? That’s not normal, but you can’t really convince a middle-aged male paediatrician of that. It took a lot of doctor shopping to get someone to pay attention.

(Maria, New South Wales)

For mothers like Maria, the intrinsic knowledge about her baby’s feeding behaviour and unusual crying empowered her to ignore advice to stop breastfeeding and explore various alternatives to address her supply and breastfeeding concerns. However, Maria acknowledged that her professional skills and her financial means enabled her to do this. Her narrative exemplifies that some women turn to galactagogues to maintain agency, in response to feeling disempowered and unsupported during this stressful time.

On the other hand, some women reported that they highly valued the practical and logical information provided by health care professionals, that was based on evidence and expertise, and offered practical solutions to their feeding challenges, with galactagogues used as part of a suite of strategies to improve supply. In particular, a number of women discussed how the support provided by the Australian Breastfeeding Association (Australia’s largest breastfeeding support service) was of high quality and considered to be particularly useful.

A small number of participants also mentioned the introduction and use of galactagogues through the unsolicited advice or recommendations from friends, particularly through those who had previously experienced issues with breast milk supply themselves ‘gifting’ galactagogues, as Meri mentioned:

I remember my friends had brought me cookies and stuff, like lactation cookies as well when [daughter] was first born, because she had had supply issues and stuff. So, I’d taken them sort of at the same time [as pumping]

(Meri, South Australia)

While some did not perceive this as harmful, others felt this practice by often well-meaning peers undermined their sense of agency, by pre-empting breastfeeding difficulties.

### Determination to provide breast milk

Women’s accounts also revealed that a strong determination to provide breast milk was an important motivator to use galactagogues. For some participants, the goal was not necessarily to breastfeed, but rather to give their babies access to the nutritional, developmental and immunological benefits of their breast milk (e.g. via bottles of expressed milk). Several participants described themselves and competitive and stubborn and this influenced their determination to maintain a supply at all costs as explained by Alana (South Australia) *“I’m a bit of a type A personality*, *where I kind of just don’t give up”* and Tara (Victoria) *“we just sort of stuck at it because I’m a bit stubborn”*.

However, for some women, their determination to keep feeding suggested that they prioritised one outcome–boosting milk supply–above all others. Prioritising this outcome led to explanations such as “just giving it a go”, “it can’t hurt”, “I can just stop”, “I have nothing to lose”, looking to “give my body an extra boost” or “buying more time”. For some, this determination to maintain an adequate breast milk supply impacted on their sense of agency. Women talked about feeling “desperate” to try anything having exhausted all other options. Mandy explains her “desperation” in her determination to try anything meant she had little choice:

It was either that [take domperidone] or not continue, I think. Yeah, I was desperate for something to work

*(Mandy, South Australia)*.

The desperation and determination arose as participants were often contemplating discordant options—either take the drug or cease breastfeeding. The option to cease breastfeeding or pumping was problematic as many had a strong opposition to infant formula, and in doing so they were left with no alternatives other than galactagogues, regardless of consequences. The strong underlying determination explained why women who were so focused on their supply used a galactagogue prophylactically or followed recommendations to start very early postpartum (e.g. before 7 days), particularly those who had previous issues with supply.

### Broader dimensions of decision-making

The broader dimensions reflect both internal and embodied aspects that were described as important to decision-making, as well as external contexts in which women were making decisions.

Factors related to a *Maternal emotional wellbeing* were frequently discussed as important, in variety of ways. Many women reported high levels of anxiety, emotional turmoil and stress whilst managing their milk supply, that were not necessarily addressed by postnatal support, and affected their ability to make informed choices about galactagogues. Some women reported making decisions about breastfeeding and galactagogues in the context of extreme depressive symptoms (including some reporting postnatal depressive disorder) which they felt influenced their breastfeeding goals and subsequent decision-making. As participant Alana explained, her experiences and attempts to boost and maintain her supply were extremely emotional and stressful and influenced her ability to rationalise at the time:

I look back, and the emotional and psychological pressure I put on myself for the six months with the exclusive expressing and everything really was not healthy, and I look back now and think, oh, god, I should have stopped [expressing] way sooner, for my mental health. In the moment, when you’re emotional and you’ve got all those hormones flowing through your body, you have this beautiful new baby that you want to give everything to, you just–you get a bit irrational, really

*(Alana, New South Wales)*.

Others discussed the ways in which previous mental health and trauma experiences (including discussions around history of abuse, disordered eating and loss and grief) heightened their emotional and stress responses when breastfeeding challenges arose and influenced the importance they placed on providing breast milk, creating a heightened desire to address these challenges.

*Social norms and pressures* that promote breastfeeding as the ‘right and normal choice’ for infants also factored into decisions about persisting with breastfeeding. Some women sought galactagogues to avoid judgement arising from family and cultural expectations around feeding, reflecting involvement of partners and mothers, mothers-in-law and sisters, as well as interactions with peers and the wider public. For example, Olivia explained how she felt judged by her mother’s expectations when she was not able to breastfeed which prompted her to consider galactagogues for support:

*My mum’s very pro–really pushed the breastfeeding, which I did myself anyway, but I felt like–she breastfed five kids, so I felt like she would have judged me, and she did, she did*.

(Olivia, New South Wales)

Many women spoke of feelings of guilt and failure when feeding challenges arose, with negative influences on their sense of agency. Apprehensions around the connotations of maternal failure played a significant role in their response to insufficient milk supply and decisions around the use of galactagogues as Beth discussed:

I felt like breastfeeding is the only thing that you’re supposed to do, and I felt like I wanted to feed, so I felt like if I couldn’t breastfeed her, I was failing, and if this medication was a thing that was going to make me be able to breastfeed, then obviously I just needed to do it

(Beth, New South Wales)

The anxiety around being perceived as a failure was also deeply woven into the fear of being depicted as a neglectful mother for not providing breast milk. The fear of being judged as negligent was heightened particularly around interactions with some health professionals when a baby was not gaining weight. For some women, use of galactagogues was prompted directly in response to external and their own *Concerns about infant development*. For example, Dominque recalled an encounter that she had at a scheduled six week check up with an infant health nurse:

*We went to have the appointment and they’re like ‘she’s not putting on weight, you have to supplement with formula now. You have to or else you’re like neglecting your child. You have to do it’ … I was actually told ‘if I didn’t do it, she would die’ was the actual sentence*.

(Dominque, New South Wales)

Concerns were particularly significant for premature or low birth weight newborns or those who were considered “failure to thrive” (a common yet problematic term to describe babies who are not meeting growth milestones). Also discussed were the babies who had been diagnosed and treated for tongue-tie (ankyloglossia), a frequently debated and controversial issue. For example, among women who didn’t respond to galactagogues, often the baby’s tongue-tie was problematised rather than breast milk supply.

Factors related to *Maternal physical health* were also influential. Several participants discussed how issues relating to fertility/subfertility (and subsequent treatments), pregnancy and birth influenced their breastfeeding outcomes and how they responded. For instance some saw separation following birth by caesarean section as interrupting establishing milk production, or spoke of the impact of a traumatic birth on both mother and baby (i.e. injuries to baby resulting in disinterest in suckling). Also discussed here were other factors including anatomical issues (milk ducts, breast tissue), thyroid function, allergies, obesity and recovery from gastric by-pass surgery which were seen as physiological barriers that could be supported by the use of galactagogues.

*Lactation history* was relevant for women having their second or subsequent child and influenced their knowledge and assumptions about breastfeeding. In particular, previous use of galactagogues factored into their decision-making through their evaluation of the effectiveness and benefits of galactagogues they had already used. In addition, based on previous experiences with supply, a minority of women decided to use galactagogues prophylactically, taken as a precaution to counteract concerns about supply with the current child.

Also pertinent were the current lactation experiences prior to considering a galactagogue. This was important as participants rarely used galactagogues as the immediate and initial response to supply concerns (with the exception those using it prophylactically), indicating they were not bypassing other recommended strategies (e.g. increased frequency of milk expression) to boost supply.

A *Desire for a guaranteed and urgent response* to feeding challenges played strongly into the women’s decision-making, with some women describing the perceived effectiveness of a galactagogue and the time it would take to be effective as critical to their decision. For some, the processes of information gathering, support and galactagogue use were occurring simultaneously (rather than allowing time for the support to elicit a change and alleviate concerns about supply) emphasising the feelings of pressure and degree of urgency influencing decision-making. This urgency resulted in many participants indicating that they were using more than one galactagogue at a time. The urgency was also related to barriers associated with access to breastfeeding support, described earlier as high demand and long waiting lists. For some women, the sense of urgency was enhanced by the desire to avoid feelings of maternal failure and stigmatising judgements about their mothering.

The issue of a guaranteed outcome was often discussed by women around the use of the supplement fenugreek. Many women explained that the uncertainty associated with fenugreek was based on anecdotal information about the possible unwanted effect it could have on reducing supply. The distinction between either boosting or reducing supply was seen as too uncertain leading many women to decide against fenugreek. As Maggie explained:

Often, a lot of the alternative medicines, I just thought, “No, I don’t believe it” or with the fenugreek, every time someone would post on this group, “I’m taking fenugreek to try and boost my supply”, there would always be a bunch of people who said, “Oh, that can also decrease your supply.” Apparently, fenugreek can go either way. So I thought, “Well, that’s enough for me to not bother.”

(Maggie, Western Australia)

Participants also discussed expectations that there would be an immediate boost in supply after taking domperidone with many anticipating a quick response. This urgency was illustrated by the women who, after not experiencing the anticipated boost to supply, increased their dosages of pharmaceutical galactagogues without medical supervision.

Many women spoke about actively weighing up perceptions of side-effects with the potential (however many perceived this as a guarantee) of domperidone boosting supply. These processes reflect varied and complex accounts reflective of a *Risk-risk trade-off*, where risks were seen to be borne by the mother in juxtaposition to her responsibility to baby. For some, the side-effects of domperidone was considered a reason for avoiding the medication, whereas others saw the potential of the side-effects as minimal, with the possibility of increasing supply far outweighing any concerns, as Deb explained:

I knew about the side-effects of [domperidone], but I was like, I was willing to risk those side-effects, you know, to be able to feed my son

*(Deb, New South Wales)*.

For other women, who decided against the use of galactagogues, their assessment of the risks and limited guarantees about effectiveness outweighed the potential benefits. For some women, their decision to avoid galactagogues (mostly domperidone) represented the end of their breastfeeding journey, sometimes contributing to feelings of being judged and pressured, but also relief and satisfaction that they had done all they could.

In considering the risk-risk trade-offs, many questioned the practice of prescribing domperidone “off-label” (the prescribing of medications outside the approved use) as a potential red flag. Others were aware that domperidone is not approved in the US and saw this as a deterrent, raising safety concerns. In contrast, some participants were not aware of potential adverse effects, explaining that they were either not told or could not recall, as illustrated by Tammy:

Facilitator: … so did the doctor talk to you about it [domperidone], or they wrote you a script and…

*Tammy: I honestly can’t remember. I’m sure he said, he would have talked to me about something, but I don’t remember having a clear conversation about what it does. I mean, he told me that it will increase my milk supply but in terms of any effects, or anything like that, I have no idea*.

(Tammy, South Australia)

The limited discussions of side-effects were interpreted as an indication that the medication was risk-free, indicative of the quality of breastfeeding support provided. On the other hand, herbal or dietary galactagogues were mostly considered innocuous (with the exception of fenugreek as discussed above) which meant they were consumed with less scrutiny of the potential risks.

In considering the quality of information they received about risks and benefits of galactagogues, women often viewed themselves as the ‘problem’ rather than inadequate breastfeeding counselling, discussing how they were unaware of the side effects because they were hormonal or in a sleep deprived ‘haze’ or ‘fog’ after giving birth. They explained that they were not able to filter information due to the stressful circumstances associated with having an unsettled baby, a baby who was not gaining weight or a premature baby in special care nursery. For many women this ‘haze’ they described impacted their ability to ask questions and assess information in order to consider the risk-benefits appropriately, indicative of a diminished sense of agency.

The *Acceptance and trust* factors associated with decision-making included reports of entrusting the authority of doctors’ suggestions, resulting in minimal considerations of alternatives. Some women who reported that they took the medication or used the substances recommended without question, indicated that they deferred or entrusted the decision to experts, as Tammy reflected in regard to her premature sons:

Facilitator:… So, when you were in conversation with your obstetrician, talking about [domperidone], did you perceive any risks with taking it?

*Interviewee: No*.

Facilitator: No? So it didn’t come up in your chats with him?

Interviewee: Yeah, I just said I’m a bit–no, I think he asked “How’s your milk going?” I’m like “I’m a bit worried about it” and he’s like “Here, take this” … I think it was that casual

*(Tammy, South Australia)*.

Women were not passive in this situation and the ways in which decisions were deferred was influenced by circumstances and priorities such as: concerns about premature babies and limitations on ability to carry out maternal activities, other caring responsibilities, high levels of anxiety, sleep deprivation, indecision, confidence, inexperience, or limited access to information or advice that resonated. As Bobbi explained:

… unfortunately like I said, I was probably in a space of just doing what people said a little bit more than question it because I wasn’t thinking as clear as I should have and he still doesn’t sleep so it’s just one of those things that you lose I suppose some capacity to think when you don’t sleep

*(Bobbi, New South Wales)*.

Whilst women valued and trusted the knowledge of health care experts, some were insistent on having more autonomy which involved them being more critical of information they collected, for example:

I don’t put too much value in word of mouth. I’m like, give me cause and effect. Where’s your study? How big’s your sample size, blah, blah, blah. You know? I don’t want to hear about how onions magically stop boob milk

(Jen, New South Wales)

Jen’s experience highlights the way some women navigated the difference between evidence and anecdote. However, for others, the separation of evidence and anecdote was not always distinct, with many piecing together information from multiple sources to gain a comprehensive overview of their options, as explained here:

I tried my hardest to go for things that are peer reviewed. Things that have solidified evidence behind it, but as you do, sometimes you just Google things and you know, well that might be true, that’s somebody’s experience, that might be right …I think that’s where I sort of put two and two together using that information

*(Meri, South Australia)*.

As Meri’s example illustrates, when information was absent or contradictory women collated pieces of information from multiple sources (both evidence-based and anecdotal) prioritising what they interpreted as practical and relevant to their own circumstance, and information that acknowledged the shared difficulties and emotional aspects of breastfeeding issues.

Although some women indicated that trust operates in the decision-making process where the authority and knowledge of doctors and health care providers is an assurance of safety, others indicated that trust could also be damaged around the inconsistency of information. This was discussed by participants who highlighted how contradictions, gaps or limited information was an impediment to decision-making. For example, some explained that the absence of information available regarding the effect of pharmaceutical galactagogues on their supply or potential for side-effects on their baby, flagging this as problematic in terms of helping them make evidence-based choices. For those who saw the knowledge of health care professionals as lacking they often privileged and trusted other women’s embodied experiences and narratives as a more relevant source of information to base their decisions on:

So the Facebook group was the biggest support because it is personal experience rather than a medical professional who might not have actually even dealt with many lactating mums

*(Anita, South Australia)*.

The *Accessibility and cost* factors that were considered included convenience, practicality and financial burden. For a number of the women, accessing various herbal galactagogues was not seen as viable because they either could not afford them or were not able to source them in a cost-effective way. When the herbal or dietary supplements were not accessible due to high cost, the pharmaceutical or homemade options were often perceived as more cost-effective, as Anita described:

In the meantime, I was also making my own booby bickies because I’m povo [poor] and can’t afford to pay $20,000 for one cookie that has only got brewer’s yeast and flaxseed meal in it anyways

(Anita, South Australia)

Although mothers like Anita found costs restrictive, other mothers considered the financial burden to be the most insignificant factor in their decision-making, as Maria explained; *“the only thing I was losing was money*, *so why not try it*?*”*.

## Discussion

This study provides insights into the complexity of factors underpinning decisions about the use of galactagogues as women navigate new and expanding motherhood. Our findings reveal three core domains influencing decision making, reflecting the availability and quality of breastfeeding support, attempts to restore or maintain maternal agency and the underlying determination to breastfeed. These domains are interrelated, such that experiences in one domain influence how women draw on the other domains to make decisions. A key finding is the range of problematic interactions women had with health care professionals regarding breastfeeding support, that resulted in feelings of being dismissed and the provision of conflicting information, that was sometimes at odds with beliefs about breastfeeding. From these experiences’ women felt undermined and some turned to galactagogues to regain agency to achieve their breastfeeding goals.

Our findings also reveal a range of broader dimensions influencing decision making which are highly individualised and interlinked with other dimensions as well as the core domains. We found decisions about galactagogues were often underpinned by high levels of stress, which were often be amplified by social norms about breastfeeding being ‘natural’ and feelings of maternal failure when difficulties arise. Interpretations of these findings suggest that some women felt they did not see the use of galactagogues as a ‘choice’, indicative of a diminished sense of agency.

The experiences of stress and resultant effects on wellbeing in this study often impinged on women’s ability to balance risks associated with galactagogue use against their own physical and mental health and the consequences of different feeding options for the health of their infant. The prioritising of the benefits to baby over mother may be interpreted as evidence that women are denying their own agency [8] as they had no choice but to take them, in order to avoid stigma and shame around not breastfeeding. However, it is critical to emphasise that the range of factors identified in this study that influence decisions about galactagogues illustrate that women are not simply docile in making decisions about using a substance to boost their supply. Instead, they are deliberating on galactagogues with regard to the consequences for their identity as a mother, often as a way to ‘restore lost agency’ [8] in the face of inadequate breastfeeding support including insufficient information about risks, in a context of high emotions, stress and sleep deprivation [8].

The findings of this study relating to the large volume of information women receive emphasises the multiple and frequently contrasting details that at times was difficult to navigate. At a time when some women admitted feeling overwhelmed, sleep deprived, hormonal and not being able to rationalise, the presentation of contradictory information complicated the *informed* decision-making process. Moreover, the inconsistent information was often not only at odds with the predominant ‘breast is best’ messaging in health care but also signals that women’s values and beliefs (including their breastfeeding goals) may not always be factored into the information and support they receive, which has been demonstrated in research in other high income country settings [36,37].

We argue that simply improving the provision and volume of information about galactagogues will not be sufficient to support women in their decision-making. This aligns with previous research regarding infant feeding decisions, which Sheehan and colleagues [38] argue can only be adequately supported when they are viewed in relation to individual experiences and immediate sociocultural contexts. We argue that support must include consideration of the moral aspects of the ‘breast is best’ messaging, and the implications that has for maternal psychological wellbeing and agency when breastfeeding difficulties are experienced. For many participants in this study, ideologies about being a good mother and breastfeeding as a moral imperative were important factors in their decision to use galactagogues and contributed to feelings of stress. These emotional aspects underscore the need for access to high quality postnatal care that includes psychological support that offers practical information and communication approaches that work to enhance or restore women’s agency [8].

The urgency and need for a guaranteed solution was exemplified by women’s practices of using multiple combinations of substances and by initiating galactagogues concurrently with other techniques. These practices may obscure the need to address possible underlying supply issues through non-pharmacological breastfeeding support strategies. As well, it may also suggest that some women have unrealistic expectations about the effectiveness of galactagogues. To address these issues, we argue that breastfeeding advice and support would be more beneficial if it contained clearer and concise information about the effectiveness of galactagogues and the reasons they are used, in order to manage expectations and avoid possible negative impacts on maternal agency, confidence, anxiety and self-efficacy, which can have negative psychological effects {Henderson, 2016, The price mothers pay`, even when they are not buying it: Mental health consequences of idealized motherhood} [36,37].

In the context of breastfeeding and postnatal support that is alarming, dismissive and judgemental, conflicting or missing information has the potential to damage trust in health professionals and may be influencing women to seek out and value alternative sources of information. Although many participants indicated their preference for evidence-based information, this may reflect the high level of education and the employment background of this particular participant cohort. Whilst some women trusted health professionals as experts, others regarded the subjective experiences of other women as ‘expert’ advice. The growing relevance, influence and access to information drawn from lived experiences and anecdotes discussed in the interviews offers an interesting contrast to the value of evidence-based information and is an important finding in this research. Anecdotes were featured in stories shared on social media, the commercial and marketing material women were exposed to and attached to gifts supplied by well-intentioned family and peers.

Existing research verifies that women are more frequently engaging with Google searches and social media specifically seeking information about breastfeeding concerns [30,39], suggesting a shift away from the influence of experts. For example, websites selling foods and herbal substances (i.e. commercial lactation cookies) feature testimonials advertising instant results, influencing women’s assumptions about how off-the-shelf products work. The value and reliance women placed on the information tied to product promotion suggests that when information and support from professionals was inaccessible, dismissive, unhelpful, contradictory or inconsistent women actively sought out and engaged with popular sources of information and support, unverified claims, and solutions that filled these gaps. The information attached to these commercial products has been carefully crafted to take advantage of these elements of confusion by offering their merchandise as a solution. As well, the gifting of galactagogues may be associated with the increased marketing, promotion and availability of galactagogue products (sold in many gourmet foods stores) that women are using to not only support each other and enhance maternal agency.

In valuing these sources of information as credible and reliable, women may also risk basing decisions on unfounded advice and information [40]. For instance, engaging with anecdote, marketing material or gifting may be encouraging women to self-diagnose or misinterpret the source of the breastfeeding issue (e.g. problematising supply rather than addressing latch). These findings indicate that there is a considerable need to address inconsistencies and evidence gaps regarding the effectiveness and safety of commonly used galactagogues, with an emphasis on the communication styles relied to deliver and support this information [38].

As the evidence presented in this paper indicates, women are not positioned as passive and are highly invested in finding ways to continue and prolong the provision of breast milk. This research has identified that, in the context of experiences associated with insufficient breast milk supply, one of the key elements in the continuation of breastfeeding is high quality, comprehensive lactation support that positively response to women’s goals and values. The importance of empowering breastfeeding support has been widely recognised and is an ongoing and urgent priority requiring increased investment to improve lactation support [1]. Ways of addressing support needs have been explored in a recent study by Blixt et al [36] who provide a comprehensive summary of the type of breastfeeding support women are specifically seeking. They outline five key categories including evidence-based care, earlier preparation for possible breastfeeding challenges during pregnancy, respectful and mutual dialogue, individual solutions to problems or concerns and practical support. The findings of this study align with the recommendations outlined by Blixt and colleagues [36], however, we offer an expansion by recommending that individualised and tailored support could also be improved by involving processes that encourage trust and shared decision-making regarding breastfeeding challenges, which may or may not include recommending galactagogue use. The shared decision-making elements would help to maintain and enhance women’s agency which this research has found is often compromised during the decision-making process.

We argue that breastfeeding support should begin with an assessment of whether milk supply is real or perceived and then ensure other measures are exhausted before considering galactagogue interventions [41]. To ensure women are exhausting non-galactagogue strategies, approaches with professionals may benefit from adopting an individually tailored, shared approach as it is considered a more bidirectional and holistic approach to decision-making [42, see also 43–46]. A shared decision-making approach would enable women to engage with practices to support breastfeeding and/or boost supply which are attuned to the complexity of their situations and individual values.

To support this approach to decision-making, we suggest that women may benefit from the design and implementation of a shared decision-making aid that could be used by health care professionals in conjunction with women [47]. According to Kennedy and colleagues [47], decision aids can help women understand terminology, options and alternatives to care, enable them to express their personal values and reduce decisional conflict. Domains and dimensions of decisions presented in this paper could be factored into the design of a high-quality decision-making aid that enables informed choice and a shared approach that eliminates the possibility of support being dismissive or judgemental. A shared decision-making approach to breastfeeding support is currently being piloted in areas of North America [48, see also 49] and based on these findings similar approaches may be beneficial if adapted in other locations, in conjunction with decision-aids as required.

Finally, as women in this study were seeking breastfeeding support and information from many different sources, there is evidence to suggest that women are not systematically engaging with or being directed to an IBCLC when problems arise [1]. IBCLC professionals are seen to be the best-practice approach to accessing breastfeeding support as they are trained based on globally recognised, evidence-based standards [50]. However, access to IBCLC in this study was mixed, in some cases participants could not say with certainty whether the lactation consultant they engaged with was an IBCLC, and others identified barriers around access including cost (for private consultations) and accessibility which they saw as limited because of high demand for these services (particularly in public health care). As Garner and colleagues [51] argue, these barriers create “discontinuity” in breastfeeding care and support where health care providers rely on others, resulting in gaps in care, inconsistent messages and poor communication. As the findings presented by Garner et al are consistent with the findings of our study, we strongly recommend an urgent investment in and mainstreaming of IBCLCs in order to ensure women receive equitable and consistent support grounded in internationally agreed on strategies. We suggest that these strategies will be more impactful if they are inclusive of a shared decision-making approach to breastfeeding challenges [50]. Due to the critical role of GPs, they may also require further training to best support women in collaboration with trained breastfeeding consultants [52].

## Limitations

The group of participants were highly educated and employed in health or health related fields (including research). Information about the household income or other social and cultural practices were not collected but the few demographic details available present a group of participants who were predominantly white, heterosexual, able-bodied group of women who reported high levels of education. This is reflective of the overall characteristics of participants in the online survey (the source of participants for this study), thus recruiting more participants from the survey would not have increased diversity of this sample.

## Conclusions

These findings shed light on the complex range of influential factors underpinning decisions to use galactagogues. For many participants, decisions were driven by a strong sense of determination to breastfeed and the need for an urgent ‘fix’, leading to use of multiple concurrent strategies to address breastfeeding challenges. Decisions occurred in a background of inconsistent and sometimes conflicting and unhelpful information and support about breastfeeding, leading some women to actively engage in an assessment of benefits and risks whereas others delegated responsibilities to a health care provider. Women often reported feeling highly stressed during this time, navigating the physiological and emotional changes that accompany motherhood, under a weight of expectation about breastfeeding being the natural and best ‘choice’ for infant feeding. As feelings of being ‘dismissed’ were a key feature of some women’s experiences with health care professionals, the findings shed light on ways that existing breastfeeding support can influence maternal agency. We suggest improvements to breastfeeding support that addressing inconsistent and judgemental messaging and incorporates shared decision-making approaches during this intensive period of early mothering is needed. Improved support strategies must also be accompanied by high quality research that addresses evidence gaps regarding the effectiveness and safety of commonly used galactagogues, to provide reliable information to assist women and health care providers during this time.

## Supporting information

S1 File (DOCX)
