# Supplementary material for: The risk-risk trade-offs: Understanding factors that influence women’s decision to use substances to boost breast milk supply
Source: PLoS One. 2021 May 3;16(5):e0249599. doi: 10.1371/journal.pone.0249599 (PMC8092651; doi:10.1371/journal.pone.0249599)
Supplement: S1 File — (DOCX) [file pone.0249599.s001.docx]

Interview Schedule/Topic Guide

Medicines and Other Substances For Improving Breast Milk Supply

**Introduction/Preamble**

Thank you very much for agreeing to take part in my study and providing informed consent. I have explained to you earlier that the aim of this project is to explore your awareness and use of medicine and other substances for increasing breast milk supply. I assure you that you will not be identified in any written report or papers generated by this study. To assist with data analysis, I will be recording our conversation. However, you can ask me to stop recording at any time. There are no right or wrong answers in this conversation. So, I am encouraging you to tell your story in your own way.

**Interviewing women**

Now I would like to start recording. Are you happy to proceed?

*[Recording will be started from below]*

Recorder turns on

**Today is ……………...........2019. I am talking to participant …………**

**Topic Guide:**

| Breastfeeding | First of all,   - Lets talk about your baby, are you feeding now and how’s it going |
| --- | --- |
| Low supply | What was your experience with your breast milk supply?  Did you ever worry about supply issues? What made you concerned? |
| Decision making | Did you ever think about taking something to boost your supply? How did you work through making this decision  Who did you talk to about this? What advice did they give you?  What info was more helpful than others?  Was there anything that didn’t sit well with you regarding information received?  Was there anything that was unhelpful?  How did you work out which information you got is right for you/not right?  Did you perceive any risk with taking any medication or substance?  What factors influenced your perception? |
| Medication or Substance Use to Boost Milk Supply | What medication/substance did you end up using?  What was the most important factor that led you to use X?  If supply was not an issue – why did you take it?  How did you work out what to do?  What happened after you starting taking it?  Did the result force you to change anything?  What factors influenced how long you took it for?  If you had your time over, would you follow the same process and use the same thing/s?  What factors influenced whether you would recommend it to someone else? |
| General | Do you feel your views are different to other peoples?  Given everything we have talked about, were you happy with the decisions you made around taking/not taking? |
| Can I talk to you again if I have some queries? | |

**Thank you very much for your participation in this study.**

Recorder turns off.
